# Tunable network architecture in a hydrogel with extreme vibration damping properties

**DOI:** 10.1038/s43246-025-00857-5

**Published:** 2025-07-11

**Authors:** Graham J. Day, Qicheng Zhang, Chrystel D. L. Remillat, Gianni Comandini, Adam W. Perriman, Fabrizio Scarpa

**Affiliations:** 1 Bristol Composites Institute, School of Civil, Aerospace and Design Engineering (CADE), University of Bristol, BS8 1TR Bristol, UK; 2 School of Cellular and Molecular Medicine, University of Bristol, Bristol, BS8 1TD UK; 3 Centre for the Cellular Microenvironment, Division of Biomedical Engineering, James Watt School of Engineering, Advanced Research Centre, University of Glasgow, Glasgow, UK; 4 Research School of Chemistry and John Curtin School of Medical Research, Australian National University, GPO Box 334 Canberra, ACT 2600 Australia

**Keywords:** Gels and hydrogels, Mechanical engineering

## Abstract

Damping technologies aim to control the loads and deformations generated by ambient or forced vibrations in structures and machineries used in transport applications and construction. Traditionally, the materials used in damping devices are of fossil origin, but viscoelastic biobased resources are an alternative source of damping materials. Here, we develop an alginate-based hydrogel system with diverse porosity topologies by including poloxamer 407 as a sacrificial porogen at varying concentrations. Vibration transmissibility tests and dynamic mechanical analysis reveal these gels exhibit loss factors between 16% and 28% in the 100–300 Hz frequency range and that the dynamic modulus increases over an order of magnitude compared to the static modulus, reaching approximately 3 MPa. The visco- and poroelastic and pneumatic-like effects from the tunable porous structures contribute significantly to this damping effect. Furthermore, these hydrogels are biosourced and biodegradable, providing a sustainable alternative to conventional fossil-based damping materials.

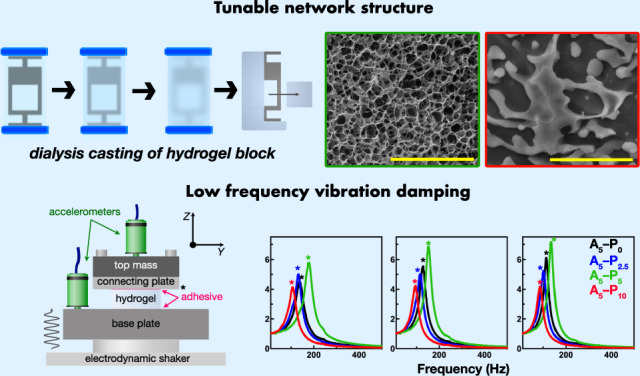

## Abstract (web summary)

Hydrogels are useful structural materials due to their viscoelasticity, which provides supportive and dissipative properties. Here, a dialysis–casting method was used to fabricate alginate hydrogels, using poloxamer 407 as a porogen, with tunable porosity topologies and high damping properties.

## Introduction

Hydrogels are hydrophilic three-dimensional polymeric scaffolds extensively used in the cosmetic industry, pharmaceutics, and in biomedical engineering as tissue scaffolds for regenerative medicine^1–3^. More recently, hydrogels are being investigated as structural materials due to their viscoelasticity, where they can provide both supportive and dissipative properties. Vibrations are a subset of dynamic loads that are critical to the structural integrity of machinery, airframes, and structures used in transport applications and construction. Vibration damping technologies have been developed during the last eight decades for the suppression, alleviation, and control of loads and deformations generated by ambient or forced vibrations^4,5^. Damping technologies make use of the synergy between the dissipative and viscoelastic properties of the constituent materials and the substrates that support those materials systems. Most materials traditionally used in damping technologies are of fossil origin, and include thermoset and thermoplastic polymers, as well as elastomers and rubbers^6^. However, biobased materials offer an alternative approach in fabricating devices for vibration damping, as well as being more sustainable and reducing the environmental impact of those materials. Synthetic elastomers can be used to produce soft, damping materials, such as polydimethylsiloxane gels (PDMS), which have been used to fill a piezoelectric porous skeleton and generate three-dimensional composites with significant damping capabilities^7^. These composite materials exhibited loss factors of around 20% within 1–20 Hz sweeps performed with dynamic mechanical analyzers (DMA). Polyurethane-carbon nanotube-PDMS gel composites have also exhibited loss factors over 24% during vibration transmissibility tests within the 50–600 Hz range^8^. The viscoelastic properties of hydrogels are extensively evaluated because of their importance in rheological and bioprinting applications. The damping capacity of hydrogel systems is also cited as a proof of their energy absorption properties under large quasi-static and dynamic loadings^9,10^. Hydrogels can be produced solely of or in composite with biobased and biodegradable compounds and offer very interesting performance in terms of damping capacity^11^. For example, alginate–silicon nitride/polyvinyl alcohol exhibited recoverable energy dissipation when subjected to approximately 3–6 J of kinetic energy in drop-tower tests^12^, and there are many examples of alginate and protein-based hydrogels employed in mechanobiological studies owing to their tunable viscoelastic properties, as assessed by rheology^13,14^. Additionally, chitin-based hydrogels and physically or chemically cross-linked hydrogels hosting starch granules can protect eggs or fruit from impact, owing to shear-thickening properties^15^.

The characterization of hydrogel materials from a purely vibration damping perspective has received, however, limited attention. Polyacrylamide (pAAM) and PDMS gels exhibited viscous damping ratios of 2–3% and 8%, respectively, using a bending rig with base acceleration excitation^9^. A high elastic modulus (1–2 MPa) polyacrylic acid–carboxymethyl cellulose hydrogels exhibited better vibration damping performance over gels with low elastic modulus (0.1 MPa), in qualitative studies^16^, and the damping ratio of gelatin discs increased with matrix concentration, as measured by Laser–Doppler vibrometry (LDV) in the 100–600 Hz range^17^. Additionally, alginate–pAAM hydrogels with tuneable energy dissipation properties, as measured by tensile hysteresis, were assessed briefly using vibration transmissibility measurements and exhibited a resonant frequency of 70 Hz, but no other information was inferred^18^.

Alginate/poloxamer systems have shown some promise as bioink platforms for tissue engineering and scaffolding, also in view of their bioprinting capabilities^19,20^. Alginate is a polysaccharide naturally found in brown algae and is composed of polymeric β-D-mannuronic acid (M-block) and α-L-guluronic acid (G-block) connected by 1,4-glycosidic bonds^21^. Alginate gels are formed by cross-linking the acid groups with multivalent metal ions. Poloxamers, also known as pluronics, are synthetic, thermosensitive triblock copolymer, which undergo a solution-to-gel (sol–gel) transition above a critical temperature, characterized by a micelle-driven microphase^22,23^. Previous work has employed poloxamer as a sacrificial viscosifier of hydrogels during 3D bioprinting by extruding onto a plate heated to a temperature above the critical sol–gel transition^19,24,25^. Subsequently, the extruded gel is supported by the microphase separation between the poloxamer and hydrogel polymer. When the hydrogel is cross-linked, the poloxamer transitions back to a solution and vacates the final cross-linked hydrogel, leaving behind a porous structure. Consequently, in this work we hypothesize here that the multiscale porosity of alginate hydrogel systems can be tailored by varying the concentration of the sacrificial poloxamer, leading to different hydrogel network architecture that will elicit differing mechanical performance and poroelastic effects, impacting the transport properties of the solvent through the polymeric network of the hydrogels and contributing to energy dissipation and influencing the vibration damping properties of the resulting porous materials^26–28^. We fabricate sets of alginate hydrogels of increasing alginate concentration and initial poloxamer concentrations through a method involving simultaneous casting and dialysis. The relationship between the pore size, alginate network structure, and compressive mechanical properties are investigated, and the experimental data is validated with a Mooney–Rivlin model of hyperelasticity. Finally, we subject the most rigid alginate/poloxamer hydrogel system to vibration transmissibility experiments to quantify their vibrational damping properties, which, to the best of our knowledge, has been not previously done with hydrogels. Notably, the dynamic stiffness of the porous alginate hydrogels dramatically increases when subjected to vibration loading, up to values close to 3 MPa, and they exhibit loss factors of approximately 0.28 within the 100–300 Hz range.

## Results and discussion

### Hydrogel fabrication

**Fig. 1:**
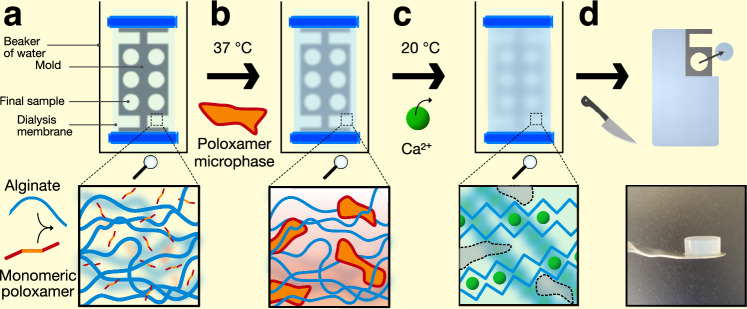
*Schematic of dialysis-casting the hydrogels.* **a** Sodium alginate polymer (blue lines) was mixed with monomeric poloxamer 407 (red–orange lines) in water at room temperature and added to a dialysis tubing membrane (light blue) containing a plastic mold (dark grey), and then the dialysis tubing membrane was clipped shut (dark blue). **b** The membrane was submerged in water at 37 °C to induce a sol–gel transition of the poloxamer, forming a microphase (red–orange shapes). **c** Calcium chloride was added to the water exterior to the dialysis membrane tubing to cross-link the alginate with Ca^2+^ ions (green circles). The solution was gradually cooled to 20 °C to reverse the sol–gel transition of the poloxamer, so the poloxamer could diffuse out of the membrane as Ca^2+^ diffused in. **d** Finally, after the alginate was fully cross-linked inside the dialysis membrane, excess hydrogel was cut away from the mould and samples of the desired dimensions were removed.

Alginate hydrogels with tunable porosity were formed by adding increasing quantities of poloxamer 407 to alginate solutions prior to cross-linking them. The G-blocks of alginate can be cross-linked by divalent Ca^2+^ to form classic ‘egg-box’ structures (Supplementary Fig. 1)^21^. Poloxamer is an amphiphilic ABA-type triblock co-polymer composed of a hydrophobic polypropylene oxide unit flanked by two hydrophilic polyethylene oxide units (Supplementary Fig. 1). Upon heating to 37 °C, the phase behaviour of the poloxamer 407 changes and it undergoes a sol-gel transition owing to dehydration and micellization of the polypropylene oxide blocks, forming a gel^29^. This transition can be exploited when mixing with alginate to form a microphase separation prior to cross-linking the alginate around the poloxamer microphase, leaving behind porous structures when the poloxamer is removed^19^. To form the hydrogel, the alginate and poloxamer are initially mixed into a homogenous solution in water, below the sol-gel transition temperature of the poloxamer. Then, the solutions were incubated at 37 °C to induce the sol-gel transition and drive microphase separation of the alginate network and poloxamer phase. Then, divalent calcium ions (Ca^2+^) were added to cross-link the alginate chains, whilst the microphase separation was still present. The solutions were removed from 37 °C to 20 °C, so the poloxamer could re-solubilize and exit the cross-linked alginate gel. Hydrogel samples of defined and reproducible dimensions were required for the compression and vibration transmissibility experiments, and the process to form them was initially conducted in specifically shaped molds to casted them in defined dimensions. However, initial attempts to cross-link the casted gels with Ca^2+^ proved difficult, as the Ca^2+^ could not diffuse evenly through deep samples, resulting in uneven or incomplete cross-linking. To solve this issue, a method involving casting the hydrogels inside dialysis membranes was conceived. 3D printed plastic molds, that were open on two sides, were inserted into a semi-permeable dialysis membrane along with the homogeneously mixed alginate/poloxamer solution (Fig. 1a). The sealed membrane was then placed inside a beaker of water and incubated at 37 °C to induce the sol-gel transition of the poloxamer (Fig. 1b). During this time, the alginate and poloxamer were thought to de-mix into a multi-gel system. Subsequently, Ca^2+^ was added to the external water as the solution was cooled (Fig. 1c), and could diffuse into the dialysis membrane and cross-link the alginate under confinement around the mold from all angles, decreasing the depth required for the ions to travel and reducing the effects of uneven cross-linking, whilst the sacrificial poloxamer could solubilize in response to the lower temperature and diffuse out of the membrane. Finally, excess hydrogel could be cut away from the mold to liberate hydrogel samples of defined dimensions (Fig. 1d), before the solubilized poloxamer phase was washed away, as previously described^19^. Two molds were designed: one to produce pucks (12 mm in height × 5 mm diameter) for compression testing (Supplementary Fig. 2); and blocks (30 × 30 × 15 mm) for vibration transmissibility experiments (Supplementary Fig. 3). The bars at the ends of the moulds indicated where the clips would be placed to seal the membrane, to ensure the volume of the membrane was constant across samples.

Using this process, a range of hydrogels with systematically increasing alginate (A) and poloxamer 407 (P) compositions were produced to investigate the effect porosity had on the material stiffness. Increasing the alginate concentrations was expected to increase the material stiffness by increasing the network density and cross-linking concentration, whereas increasing the poloxamer weight fraction was expected to increase the pore size by increasing the microphase separation effect. Three alginate concentrations were chosen: 1.25, 2.5, and 5.0 wt%, giving A_1.25_, A_2.5_, and A_5_, respectively. The structure and porosity of each alginate variant was modified by forming the hydrogel with increasing concentration of poloxamer, at 0.0, 2.5, 5.0, or 10.0 wt%, giving P_0_, P_2.5_, P_5_, and P_10_, respectively, and 12 unique gel compositions. For example, a 5 wt% alginate hydrogel formed with 2.5 wt% poloxamer is hereby referred to as A_5_–P_2.5_.

### Stiffness related to microstructure

**Fig. 2:**
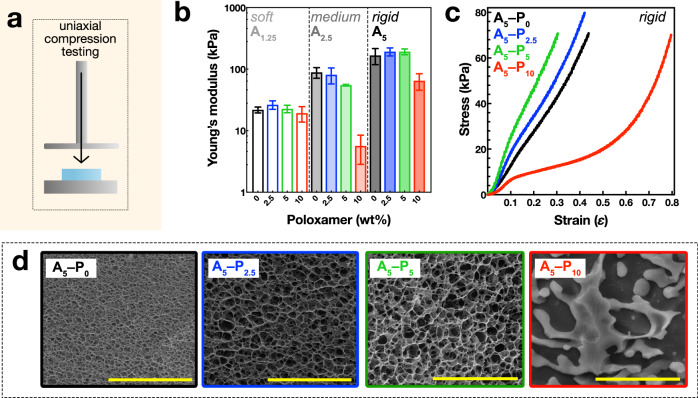
*Mechanical and structural analysis of porous alginate hydrogels.* **a** Schematic of the unconfined uniaxial compression test. **b** Young’s moduli from compression tests of soft A_1.25_ (1.25 wt% alginate; left), medium A_2.5_ (2.5 wt% alginate; middle), and rigid A_5_ (5.0 wt% alginate;, right) hydrogels. Poloxamer concentration indicated as 0.0 (black), 2.5 (blue), 5.0 (green), and 10.0 wt% (red) bars. Error bars represent range.c **c** Stress–strain curves for the rigid A_5_ hydrogels across all Poloxamer concentrations. **d** Cryo-electron micrographs of fracture surfaces of the rigid A_5_ gels with varying poloxamer concentrations. Scale bars = 10 µm.

The effect of the alginate and poloxamer concentrations on the stiffness of the hydrogels was investigated via uniaxial quasi-static compression testing. The stiffnesses of the hydrogels increased with alginate concentration (formed with no poloxamer), as the compressive Young’s modulus (*E*) increased from 21.9 ± 2.4, 88.7 ± 17.4, and 167.4 ± 49.0 kPa for the A_1.25_–P_0_, A_2.5_–P_0_, and A_5_–P_0_ hydrogels, respectively. (Fig. 2b, black bars). This stiffness increase was due to strengthening the alginate network with higher concentrations, leading to increased cross-linking density and a network of thicker fibers^30^. For the soft alginate A_1.25_ group, no significant variation in the stiffness of the hydrogels was observed with the addition of poloxamer (Fig. 2b, left section), probably because the alginate concentration was too low to form a multigel system with the poloxamer. For the medium stiffness A_2.5_ gels, *E* decreased from 100 to 10 kPa with increasing poloxamer (Fig. 2b, middle section). The rigid alginate A_5_–P_0_, –P_2.5_, and –P_5_ hydrogels generally increased in stiffness, with increasing *E* = 167.4 ± 49.1, 192.6 ± 28.9, and 192.4 ± 20.7 kPa, respectively, although not significantly so due to large variability in the A_5_–P_0_ measurements (Fig. 2b, right section). Notably, the stiffness of the A_5_–P_10_ was much lower, at *E* = 64.8 ± 19.4 kPa, similar to the trend observed in the medium A_2.5_ gels. Inspection of the stress–strain curves revealed the A_5_–P_0_, –P_2.5_, and –P_5_ were linear up to approximately 30% strain, then started to exhibit strain stiffening behavior above 30% strain (Fig. 2c). In contrast, the stress–strain profiles of the A_5_–P_10_ hydrogels were very different—stress did not increase much until >50% strain, then they exhibited strain-stiffening behavior (Fig. 2c). When handling these hydrogels, the first three compositions were rubbery and solid, but the A_5_–P_10_ was sponge-like and exuded much water upon deformation.

Next, the effect of the poloxamer porogen on hydrogel network structure was assessed in the A_5_ group by imaging fracture surfaces of flash frozen samples via cryo-electron microscopy, to preserve the structure of the hydrated state as much as possible. The micrographs of the A_5_–P_0_, –P_2.5_, and –P_5_ hydrogels revealed tightly organized, three-dimensional networks with pore sizes visibly enlarged in the hydrogels formed with poloxamer (Fig. 2d). Measurements of the pore size diameters revealed a significant increase from 0.6 ± 0.2 µm to 1.1 ± 0.3 and 1.3 ± 0.4 µm when poloxamer porogen was present during hydrogel fabrication (Supplementary Fig. 5), confirming that poloxamer can be used to adjust the pore size of alginate hydrogels. In the sample containing the highest porogen concentration, A_5_–P_10_, these intricate structures were absent and instead the alginate network appeared biphasic, composed of dense regions of alginate separated by large voids. This loss of structure likely occurred due to the formation of a hierarchical pore structures due to the high porogen concentration, which prevented a continuous alginate network from forming, thus explaining the loss of mechanical strength observed in compression testing. Overviews of the fracture surface reveal that the hydrogel structures appear to be homogeneous (Supplementary Fig. 4), although inhomogeneities owing to a diffusion gradient of Ca^2+^ into the center of the hydrogel may have been occurred and that there may be differences in structure at small length-scales. Previous reports have suggested that using high concentrations of Ca^2+^ (>340 mM) to cross-link alginate can reduce heterogeneities between the outer and inner portions of the hydrogel^31^, less than the 500 mM Ca^2+^ used here. Additionally, the dialysis-based cross-linking proceeded whilst stirring and for a sufficient time (18 h) to allow the solutions to equilibrate.

### Mooney–Rivlin model of hyperelasticity

The data from the compression testing was modelled to reveal it fit a two-parameters Mooney–Rivlin model of hyperelasticity^32^ (**Equation 1**):

$$\sigma =\left(2{C}_{01}+\frac{2{C}_{10}}{\lambda }\right)\left({\lambda }^{2}-\frac{1}{\lambda }\right)$$

**Equation 1** Two-parameters Mooney-Rivlin hyperelastic relation between the uniaxial stress *σ*, the parameters C_01_ and C_10_ and the elongation *λ*.

**Fig. 3:**
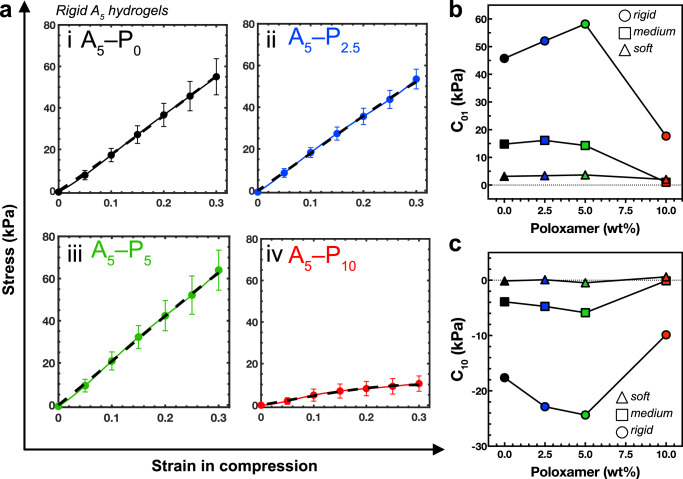
*Mooney–Rivlin model of hyperelasticity.* **a** The quasi-static uniaxial compression data was fitted to a Mooney–Rivlin model of hyperelasticity. The displayed curves correspond to the rigid 5 wt% alginate hydrogels with poloxamer at (i) 0 wt%; (ii) 2.5 wt%; (iii) 5 wt%; and (iv) 10 wt%. Solid lines with circles indicate the experimental data and the dashed lines indicate the Mooney–Rivlin model. Error bars indicate range. Please refer to the Supplementary Fig. 6 for the fits of the soft 1.25 wt% and medium stiffness 2.5 wt% alginate hydrogels. **b** The C_01_ parameter of the Mooney–Rivlin model for the 1.25 wt% alginate (Δ, soft), 2.5 wt% alginate (□, medium), and 5.0 wt% alginate (◯, rigid) at increasing Poloxamer concentrations of 0 wt% (black), 2.5 wt% (blue), 5.0 wt% (green), and 10.0 wt% (red). **c** The C_10_ parameter of the Mooney–Rivlin model, as in (**b**). Refer to supplementary Table 2 for all C_01_ and C_10_ values.

The hydrogel systems show typical Drucker-type stability characteristics, with C_0_ always positive and the total of the two parameters higher than zero^33^. The compressive stress–strain curves also exhibit no inflection point (Fig. 3a; Supplementary Fig. 6), indicating that two- or three-parameter Mooney–Rivlin models are sufficient to simulate these alginate/poloxamer systems. The relationship between the Mooney–Rivlin models and the material’s Young’s modulus is given by $6\left({{\mbox{C}}}_{01}+{{\mbox{C}}}_{10}\right)$^34^, which aligns with the actual Young’s moduli measured during the tests (Fig. 2). The C_01_ parameter is particularly sensitive to alginate content, peaking at 5 wt%. The highest magnitudes of the Mooney–Rivlin constants are observed for the 5 wt% alginate and 5 wt% poloxamer combination (Fig. 3b, c; Supplementary Table 2). This supports the findings from the quasi-static Young’s modulus tests, indicating that this specific composition of alginate and poloxamer enhances compressive stiffness by up to 30% at strain.

### Porous hydrogel with significant vibration damping properties

**Fig. 4:**
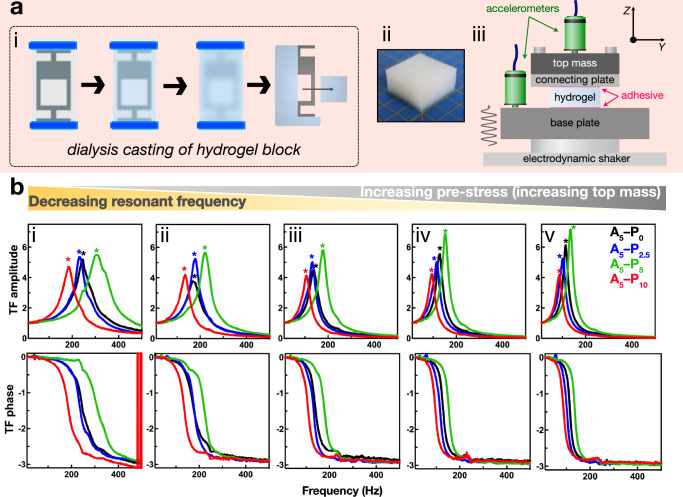
*Vibration transmissibility properties of Α*_*5*_*hydrogels.* **a** i: Hydrogel block for vibration transmissibility experiments were prepared via dialysis [see Fig. 1a–d]. ii: Optical image of a hydrogel block of dimensions 30 × 30 × 15 mm. Yellow grid demarcates 1 cm. iii: Schematic of the experimental set-up on an electrodynamic shaker. The hydrogel block was affixed between the base plate and connecting plate with alginate tray adhesive (magenta). A top mass could be attached to the connecting plate via screws, and increasingly heavy top masses could be interchanged to apply increasing pre-compressive strain to the sample prior to vibration. Accelerometers (green) were connected to the base plate and top mass to measure the amplitude of mechanical waves before and after transmission through the hydrogel. **b** Vibration transmissibility data. Representative transmissibility factor (TF) amplitude curves of A_5_–P_0_ (black), A_5_–P_2.5_ (blue), A_5_–P_5_ (green), and A_5_–P_10_ (red) hydrogels with the corresponding TF phase curves (bottom row). Asterisks indicate resonant frequency. Measurements were taken under five levels of pre-compression due to increasing load with the top mass: i) 32.44 g; ii) 64.25 g; iii) 111.10 g; iv) 160.43 g; and v) 209.13 g.

The A_5_ hydrogels were subjected to vibration transmissibility tests following an ISO 10819:2013 standard approach to explore their dynamic mechanical properties, determine their potential for mechanical energy dissipation, and investigate the effect of porosity on these properties. From such experiments, the dynamic modulus (*E*_*d*_) and dynamic loss factor (*η*_*d*_), can be determined. Hydrogel samples were prepared via the dialysis-casting method, as described above, to provide cuboid blocks with dimensions 30 × 30 × 15 mm (Fig. 4a, i–ii; Supplementary Fig. 3). To perform the vibration transmissibility experiments, a hydrogel block was mounted onto an electrodynamic shaker, sandwiched between an aluminum baseplate and a ‘connecting’ plate (Fig. 4a, iii). Additional plates of increasing mass could be systematically installed on top of the connecting plate to pre-stress the hydrogel blocks. The masses of these pre-stressing plates (including the first connecting plate) were 32.44 g, 64.25 g, 111.10 g, 160.43 g, and 209.13 g (Supplementary Fig. 7). Finally, the hydrogel samples experienced low frequency seismic vibration in the 0–1000 Hz range, and accelerometers attached to the baseplate and top mass recorded the vibration wave amplitude as a function of acceleration, across five increasing acceleration rates (1.2–5.9 m·s^−2^), with differences between the measurements of the two accelerometers corresponding to damping of vibration energy as the mechanical waves passed through the hydrogel (Supplementary Movie 1). There was a focus on small amplitude deformations in a low strain range of approximately 0.3–3%. The ratio of these amplitudes was calculated to determine the transfer function (TF; Fig. 4b top row)^26^, with the peak amplitude of the transfer function corresponding the resonance frequency (*ω*_*n*_) of the hydrogel block. Given that the stiffness of the sample is directly related to *ω*_*n*_ and peak amplitude of the TF, the dynamic modulus (*E*_*d*_), loss modulus (*E*_*l*_), and loss factor (*η*) could be determined (**Equations 3–6**) for each of the A_5_–P_0_, –P_2.5_, –P_5_, and –P_10_ hydrogels, and these values were compared across the five different levels of pre-stress and the five acceleration rates. Across all measured acceleration rates, *E*_*d*_ was invariable (Supplementary Fig. 8), indicating the hydrogels exhibited acceptable linearity in the vibration amplitude range applied.

**Fig. 5:**
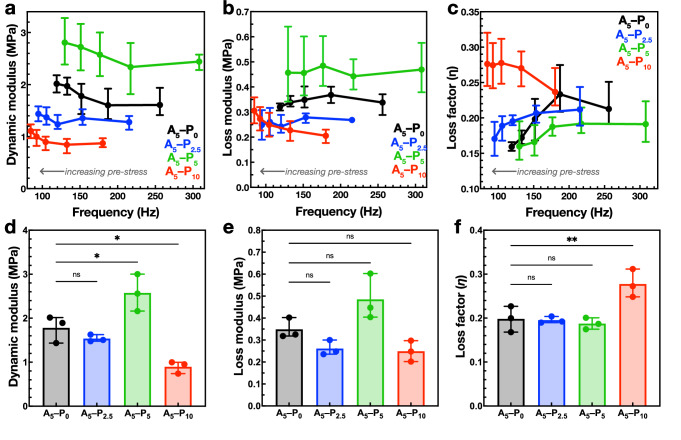
*Vibration damping properties of the A*_*5*_*hydrogels.* **a** Dynamic modulus of the A_5_–P_0_ (black), A_5_–P_2.5_ (blue), A_5_–P_5_ (green), and A_5_–P_10_ (red) hydrogels, plotted at resonant frequency. **b** The corresponding loss modulus of the A_5_ hydrogels. **c** Calculated loss factors (*η*) for the A_5_ hydrogels. **d–f** The dynamic modulus, loss modulus, and loss factors of the hydrogels measured under intermediate pre-stress (top mass = 111.10 g) at intermediate base acceleration rate (3.0 m·s^−2^). Error bars in (**a–c**) correspond to range, *n* = 3. *p < 0.05; **p < 0.01.

Comparing the peaks of the transfer functions of the four A_5_ hydrogels, as the pre-stress increased, the *ω*_*n*_ decreased with increasing pre-stress (Fig. 4b, i–v). Compared to the A_5_–P_0_ control, A_5_–P_2.5_ exhibited negligible difference (black and blue lines, respectively), but the transfer function peaks of the A_5_–P_5_ hydrogel (green) were of higher frequency and amplitude. For example, with a top mass of 111.10 g (Fig. 4b, iii), the resonance frequency for the control A_5_–P_0_ hydrogel was 175 Hz and peaked at 4.5, whereas for the more porous A_5_–P_5_, the resonance frequency was 200 Hz and peaked at 6. In contrast, the transfer function of the A_5_–P_10_ hydrogels typically exhibited lower resonance frequencies with lower TF amplitudes, compared to the other hydrogels (Fig. 4b, i–v, red line). Given that resonance frequency is directly related to stiffness (**Equation 4**), the dynamic modulus, *E*_*d*_, could be determined from these data. In each of the A_5_ hydrogels, an increase in *E*_*d*_ correlated with increasing pre-stress (and decreasing resonant frequency), likely due to higher dynamic loading (Fig. 5a; Supplementary Fig. 9). Importantly, the dynamic moduli were found to be approximately >10–20-fold greater than the corresponding Young’s moduli measured under quasi-static conditions (Table 1). For example, in the A_5_–P_0_ hydrogel, under the lowest degree of pre-stress, *E*_*d*_ was 1.6 MPa, whereas the Young’s modulus measured in compression testing was 160 kPa. As the degree of pre-stress increased, so too did *E*_*d*_, up to approximately 2 MPa at 3% dynamic strain. Similar trends were observed for all the hydrogels. The A_5_–P_5_ hydrogel exhibited an initial *E*_*d*_ of 2.5 MPa at resonance with low levels of pre-stress, which increased to 2.8 MPa, significantly higher than the control (Fig. 5d). DMA tests on the A_5_–P_5_ hydrogels conducted in the 100–130 Hz frequency range found similar dynamic storage modulus values between 2.3 and 2.5 MPa, with *tan δ* values from 0.15 to 0.18 (Supplementary Fig. 10), in alignment with the vibration transmissibility experiment. The A_5_–P_10_ hydrogel also stiffened at resonance, exhibiting an *E*_*d*_ of 0.9 MPa that increased to 1.1 MPa under increased pre-stress. The loss of the repetitive network structure led to a lower dynamic modulus compared to the other hydrogels (Fig. 5a). The dynamic modulus of the A_5_–P_2.5_ hydrogel exhibited a non-significant difference to the A_5_–P_0_ a sample (Fig. 5d), but did not exhibit intermediate values between A_5_–P_0_ and A_5_–P_5_, as expected (Fig. 5a–c). Compared to the control hydrogel, the mass and volume of the A_5_–P_2.5_ hydrogel blocks were insignificantly different, but trended such that the density was significantly lower (Supplementary Fig. 11). We postulate that the underlying reason for this phenomenon may be associated with mixing between the alginate and poloxamer 407 preventing efficient poloxamer microphase separation, although the poloxamer concentration (2 mM) was higher than the critical micelle concentration (0.3–0.8 mM) during dialysis–casting^35^.

**Table 1:** Comparison of the mechanical properties of the rigid A_5_ hydrogels

	Young’s modulus, *E* (MPa)	Dynamic modulus, *E*_*d*_ (MPa)	Loss modulus, *E*_*l*_ (MPa)	Loss factor, *η*
A_5_–P_0_	0.17 ± 0.05	1.78 ± 0.31	0.35 ± 0.05	0.20 ± 0.03
A_5_–P_2.5_	0.19 ± 0.03	1.54 ± 0.08	0.26 ± 0.03	0.20 ± 0.01
A_5_–P_5_	0.19 ± 0.02	2.57 ± 0.42	0.49 ± 0.10	0.19 ± 0.01
A_5_–P_10_	0.06 ± 0.02	0.90 ± 0.14	0.25 ± 0.05	0.28 ± 0.03

The Young’s moduli are taken from quasi-static uniaxial compression tests (see Fig. 2b), and the dynamic properties are taken from the vibration transmissibility tests with a pre-stress applied by a top mass of 111.10 g and a base acceleration rate of 3.24 m·s^−2^.

The loss modulus from the vibration transmissibility tests trended downwards with increasing pre-stress in the A_5_–P_0_ hydrogel from a peak of 0.4 to 0.32 MPa (Fig. 5b). Whilst the loss modulus of the stiffer A_5_–P_5_ hydrogel was linear, the soft A_5_–P_10_ sample increased from 0.2 to 0.3 MPa. Consequently, the loss factor (*η*; the ratio of loss modulus to dynamic elastic modulus), describing the proportion of the mechanical energy dissipated, exhibited a different trend (Fig. 5c). In the control alginate-only A_5_–P_0_ hydrogel, the loss factor decreased steeply under higher pre-stress (as the resonant frequency decreased) from 0.24 to 0.16. The trend of the A_5_–P_2.5_ hydrogel was similar. The stiffer A_5_–P_5_ hydrogel exhibited lower loss factors which plateaued at low pre-stress at 0.19 and decreased to 0.16 at higher pre-stress. Interestingly, the loss factor of the A_5_–P_10_ hydrogel was higher and increased from 0.24 to a plateau of 0.28 at higher pre-stress and was significantly higher than the A_5_–P_0_ hydrogel (Fig. 5f).

Accordingly, the architecture of the hydrogel network played an important role in influencing the poroelastic and viscoelastic damping mechanisms of these hydrogels. The high dynamic moduli and loss factors observed in these porous hydrogels were in part due to the pneumatic and hydrostatic forces exerted through the porous network, similarly to the dissipative and strain-rate effects observed in polymeric closed-cell foams with entrapped gas^36^. The dynamic stiffening effect in these gels is remarkable. For comparison, open-cell polyurethane foams with similar static Young’s moduli (120–150 kPa) show only a 50% increase in dynamic modulus during vibration transmissibility tests^26^, and articular cartilage, a biological hydrogel, also exhibited 50% increases in dynamic modulus (~30 to ~45 MPa) in the 1–300 Hz range^37^. Comparing the A_5_–P_0_ and A_5_–P_5_ hydrogels, increasing the size of the macropores in the alginate network resulted in reinforcing the network fibers and increased their effective cross-linking density. This reinforcement resulted in a stiffer network that resisted dynamic deformation, as observed in the increased dynamic moduli. Moreover, these hydrogels are cross-linked by the divalent Ca^2+^ through non-covalent ionic bonds, which, along with the movement of water through the polymer network, contributes to the viscous component of the viscoelastic alginate network and provides a key mechanism of energy dissipation^38,39^. Indeed, the mechanical properties and energy dissipation of alginate can be altered using cross-linking ions of different ionic strengths or size^18^. During slow rates of deformation, such as in the quasi-static compression tests, these ionic bonds can dissociate and re-associate, and the alginate chains can reconfigure in response to the deformation. This mechanism is responsible for stress relaxation in Ca^2+^ cross-linked alginate hydrogels experiencing a constant strain over long time periods^38^. However, under the cyclic deformation experienced during vibration, the higher rate of deformation may have impacted the Ca^2+^ binding kinetics and the ability of the chains to reconfigure, reducing energy dissipation through this mechanism. Consequently, more energy was stored in the network, rationalizing the observed high *E*_*d*_, and the trends *E*_*d*_ and *E*_*l*_ in response to larger deformations stimulated by greater degrees of pre-stress. Similar effects have been described in synthetic hydrospongels whose networks exhibited long viscoelastic relaxation times, restricting conformational changes and allowing the water-rich materials to support heavy loads^40^. In contrast, the trend between loss factor and pre-stress (decreasing resonant frequency) in the A_5_–P_10_ hydrogel was reversed: the loss factor (and loss modulus) increased under higher pre-stress (Fig. 5b, c), suggesting that a different mechanism was primarily responsible for energy dissipation. The structure of the A_5_–P_10_ hydrogel lacked a continuous network (explaining the lower dynamic modulus compared to the other hydrogels) and was composed of a biphasic features of dense alginate chunks separated by voids (Fig. 2 and Supplementary Fig. 4). It is thought that during cyclic deformation, the surfaces of these layers of dense alginate pushed against each other and dissipated energy through slip–stick friction^41^, an effect that would have been enhanced at higher dynamic strains, as observed. Moreover, the continuous voids between the alginate would have facilitated the movement of water throughout the material, dissipating energy through pneumatic or hydrostatic forces.

## Conclusions

The alginate/poloxamer systems developed in this work exhibit multiscale porosity and variable network architecture, enabling enhanced damping effects through poroelastic and viscoelastic mechanisms in the 50 Hz to 300 Hz frequency range. These frequencies correspond to low-frequency vibrations commonly encountered in machinery and transport systems, which are typically challenging to dampen using conventional elastomeric and polymeric materials^5^. The dynamic stiffening of these gels within the considered frequency range is notable, with increases of more than an order of magnitude compared to their quasi-static Young’s modulus. This significant rise in dynamic modulus, observed using a vibration transmissibility rig, is further validated by cyclic compression tests conducted with a dynamic mechanical analyzer. The alginate/poloxamer system is tunable, with both mechanical and dynamic properties sensitive to the weight fractions of the alginate matrix and the poloxamer used in the fabrication process, which influenced the final aginate network structure. The average loss factors range from 16% to 26%. A weight fraction of 5% for both alginate and poloxamer yields the stiffest hydrogels, with quasi-static moduli around 180 kPa and dynamic moduli up to 2.8 MPa. The loss factors for the A_5_–P_5_ gels range from 16% to 19%, while higher loss factors of up to 28% can be achieved with different weight fractions, although this results in lower quasi-static and dynamic moduli.

While the hydrogels developed here are produced via dialysis, the alginate/poloxamer systems can also be bioprinted^19^, providing flexible manufacturing and deposition options with additive techniques. Additionally, these hydrogels are made from biosourced and biodegradable materials, offering a sustainable alternative to conventional fossil-based damping materials on the market.

## Methods

### Production of the hydrogels

Pre-gel solutions with a mass of 50 g were produced by adding 25 g of ultra-pure water to a PP30-60ML Mix Cup (Hauschild-SpeedMixer, Germany), then adding the calculated masses of dry sodium alginate and poloxamer 407 powders (Supplementary Table 1). The powders were passed through a sieve as they were added to remove clumps. Finally, the mixture was topped up to 50 g with ultra-pure water and sealed with the lid. The solution was homogenously mixed using a dual-asymmetric centrifuge-150 (DAC150, Hauschild-SpeedMixer, Germany) at 250 rpm for 5 min at room temperature. The vessels were then left to stand on the benchtop for 15 min to give the powders time to hydrate. Finally, the pre-gel solution was mixed in the DAC150 at 2500 rpm for 10 min to fully homogenize.

A dialysis mold was inserted into a wetted dialysis tubing cellulose membrane (MWCO 14000), with a width of 43 mm (Scientific Laboratory Supplies, UK) and clipped at the bottom of the mold. The pre-gel was then poured into the dialysis tubing to cover the dialysis mold and pockets of air were removed by manipulating the membrane. The tubing was then sealed by clipping at the top of the dialysis mold. The loaded membrane was then placed into 1.5 L of pre-warmed 37 °C ultra-pure water (in a 2 L beaker) and incubated at 37 °C for 1 h to induce the sol-gel transition of the Poloxamer. To cross-link the solution and form the final hydrogel, the beaker was removed from the 37 °C incubator and the solution stirred at 200 rpm with a magnetic stir bar, taking care to avoid knocking the dialysis membrane. Subsequently, 147.01 g of calcium chloride (CaCl_2_) was added to the stirring solution followed by 0.5 L of pre-warmed 37 °C ultra-pure water, giving a final CaCl_2_ concentration of 500 mM, and left to stir at room temperature for 18 h. During this time, the hydrogel cross-linked under confinement of the dialysis membrane, whilst the poloxamer (molecular weight 12500 Da) solubilized and dialyzed out of the semi-permeable membrane.

The cross-linked hydrogel with the embedded mold were removed from the dialysis membrane and a sharp knife was used to cut away excess hydrogel from the mold and release the hydrogel sample in the desired shape. To wash out excess poloxamer 407, the final hydrogels samples were submerged under 200 mL ultra-pure water for 24 h, whilst replacing the water hourly over the first four hours.

### Cryogenic scanning electron microscopy

Cryogenic scanning electron microscopy (cryo-SEM) of the 5 wt% alginate hydrogels was conducted at the Wolfson BioImaging Facility, University of Bristol. Scanning electron micrographs were captured on a FEI Quanta 200 field emission gun SEM (Thermofisher Scientific, USA), under high vacuum. To image the samples in a condition as close to the hydrated state as possible, punches were taken from the hydrogel samples and frozen by plunging in a liquid nitrogen slurry. Then, in an environment-controlled chamber built on to the SEM, the sample was fractured and the surface sublimated for three minutes. The surfaces were coated with carbon prior to imaging.

Pore diameters were manually measured using FIJI software. Statistical analysis of the pores was performed on 290 measurements taken from three micrographs per hydrogel composition.

### Quasi-static uniaxial compression testing

Quasi-static uniaxial compression tests were carried out using an FMS-500-L2 Force Measurement System (Starrett, Australia) fitted with a 10 N load cell. Cylindrical dialysis-casted hydrogel samples, with a diameter of 12 mm and height of 5 mm, were loaded onto the bottom platen and the top platen was lowered until it contacted the top surface of the hydrogel. The samples were then compressed at a rate of 1 mm·min^−1^ until the load cell recorded a force of 8 N, at which point it stopped compressing. The Young’s modulus (*E*) each of the hydrogels was determined from the linear portion of the stress–strain curve at 5–10% strain (**Equation 2**).

$$E=\frac{{{{\rm{\sigma }}}}}{\varepsilon }=\frac{F/A}{\Delta {{L}}/{L}_{0}}$$

**Equation 2**. Determining the Young’s modulus, *E*, where *σ* is uniaxial compressive force (*F*; Newtons) per unit surface area (*A*; mm^2^) and *ε* is strain, or the change in height (*ΔL*; mm) divided by the original height (*L*_*0*_; mm).

### Vibration transmissibility tests

Hydrogel blocks of dimensions (*l* × *w* × *h*) 30 × 30 × 15 mm, were dialysis casted. The seismic vibration rig used for vibration transmissibility experiments has been previously described and characterized^26^, but will be described here. The rig consisted of a steel base plate mounted on to a vertically orientated electrodynamic shaker (LDS V406 Permanent Magnetic Shaker, LDS, UK), with the hydrogel samples mounted on the center of the base plate. The hydrogels were pre-strained by the placing a connecting plate on the top surface of the hydrogel, on to which additional masses (top mass) could be affixed with bolts. An adhesive agent (DEHP Alginate Tray Adhesive Liquid, Henry Schein, UK) was used to fix the hydrogel to the base plate and the connecting plate to the hydrogel. Two accelerometers (model 333 M07, PCB Piezotronics, USA) were screwed into the base plate and the top mass to measure the vibration signal before and after passing through the hydrogel. Holders, which could be attached on the sides of the base plate, were used to center the hydrogel, and facilitated the unscrewing and screwing of top mass plates while minimally disturbing the hydrogel. The five top masses used to pre-strain the hydrogels (including the masses of the connecting plate, accelerometer, and bolts) were 32.44 g, 64.25 g, 111.10 g, 160.43 g, and 209.13 g.

Digital white noise signals were generated by an in-house MATLAB code, which was converted to an analog signal by a NI USB-6211 data acquisition (DAQ) device (National Instruments, USA) and then amplified by a power amplifier (LDS PA100E) to drive seismic vibration of the base plate. The accelerometers measured the analog signals corresponding to vibration of the base plate (undamped, before passing through the hydrogel) or top mass (damped) and transmitted to a NI-9234 C Series Sound and Vibration Input module (National Instruments, USA), which converted the signal to digital. Finally, another in-house MATLAB code recorded the data.

The transfer function, TF(*ω*), dynamic modulus, *E*_*d*_, and dynamic loss factor, *η*_*d*_, were determined as previously described in ref. ^26^. In brief, *η*_*d*_, was obtained from a simplified seismic base vibration model, where the sample is represented by a linear spring with stiffness (real value) *k*, mass *m*_*s*_ and a hysteretic loss factor *η* (**Equation 3**).

$${{\eta }}_{d}=\frac{{a}_{m}}{\sqrt{{\beta }^{2}-1}}=\frac{M+\frac{{m}_{s}}{2}}{M+\frac{{m}_{s}}{3}}\frac{1}{\sqrt{{\beta }^{2}-1}}$$

**Equation 3**. Calculating the dynamic loss modulus, *η*_*d*_, where *α*_*m*_ is the mass coefficient[ref], *β*^2^ is the peak amplitude of the transfer function at resonance and *M* is the inertia of the top mass.

Given that resonance frequency, *ω*_*n*_, is proportional to stiffness *k* (**Equation 4**)^26^, the dynamic storage modulus, *E*_*d*_, could be calculated at resonance (**Equation 5**). Accordingly, the dynamic loss modulus, *E*_*l*_, could be calculated from *η*_*d*_ and *E*_*d*_ (**Equation 6**).

$${{{{\rm{\omega }}}}}_{n}=\sqrt{k/\left(M+\frac{{m}_{s}}{3}\right)}$$

**Equation 4**. The relationship between resonance frequency, *ω*_*n*_, stiffness, *k*, top mass inertia, *M*, and sample mass, *m*_*s*_.

$${E}_{d}=\frac{{{{{\rm{\omega }}}}}_{n}^{2}H}{A}\left(M+\frac{{m}_{s}}{3}\right)$$

**Equation 5**. Determining the dynamic storage modulus, *E*_*d*_, at resonance frequency.

$${E}_{l}={{\eta }}_{d}\times {E}_{d}$$

**Equation 6**. Determining the dynamic loss modulus, *Ε*_*l*_.

### Dynamic mechanical analyzer

Dynamic mechanical analysis (DMA) tests were performed using a DMA 850 instrument (TA Instruments). Cylindrical hydrogel samples with a diameter of 12 mm and a height of 6 mm were tested under compressive loading. Frequency sweeps were conducted incrementally from 0.1 Hz to 200 Hz in overlapping ranges (e.g., 0.1–10 Hz, 9–20 Hz, etc.), with a new pristine sample from the same hydrogel batch used for each range to limit cumulative strain. A compression clamp with a diameter of 15 mm was utilized, and the test chamber temperature was maintained at a constant 25 °C. A conditioning preload force of 0.001 N was applied prior to testing. An oscillation amplitude of 10 μm was employed to ensure elastic sinusoidal compression of the samples.

### Statistical analysis

Statistical analyses were completed using GraphPad Prism software (San Diego, USA). Data was expressed as mean with standard error (*n* = 3) and analyzed using one way ANOVA (Figure 5). *p*-values less than 0.05 were considered to be statistically significant (*p < 0.05, **p < 0.01) and *p*-values > 0.05 were considered not statistically significant.

## Supplementary information

Summplementary Information

Description of Additional Supplementary files

Supplementary Movie 1

## Data Availability

The data that support the findings of this study are available from the corresponding author upon reasonable request.
