# Supplementary material for: Tunable network architecture in a hydrogel with extreme vibration damping properties
Source: Commun Mater. 2025 Jul 11;6(1):148. doi: 10.1038/s43246-025-00857-5 (PMC12254035; doi:10.1038/s43246-025-00857-5)
Supplement: Supplementary file 1 — Summplementary Information [file 43246_2025_857_MOESM1_ESM.pdf]

Supplementary information

Tunable network architecture in a hydrogel with extreme vibration damping properties

Graham J. Day[1,2,3], Qicheng Zhang[1], Chrystel D. L. Remillat[1], Gianni Comandini[1], Adam W. Perriman[2,4] and Fabrizio Scarpa[1*]

[1] Bristol Composites Institute, School of Civil, Aerospace and Design Engineering (CADE), University of Bristol, Bristol BS8 1TR, UK

[2] School of Cellular and Molecular Medicine, University of Bristol, BS8 1TD Bristol, UK

[3] Centre for the Cellular Microenvironment, Division of Biomedical Engineering, James Watt School of Engineering, The Advance Research Centre, University of Glasgow, Glasgow G12 8QQ, UK

[4] Research School of Chemistry and John Curtin School of Medical Research, Australian National University, Canberra ACT2601, Australia

*Email: F.Scarpa@bristol.ac.uk

Supplementary figures

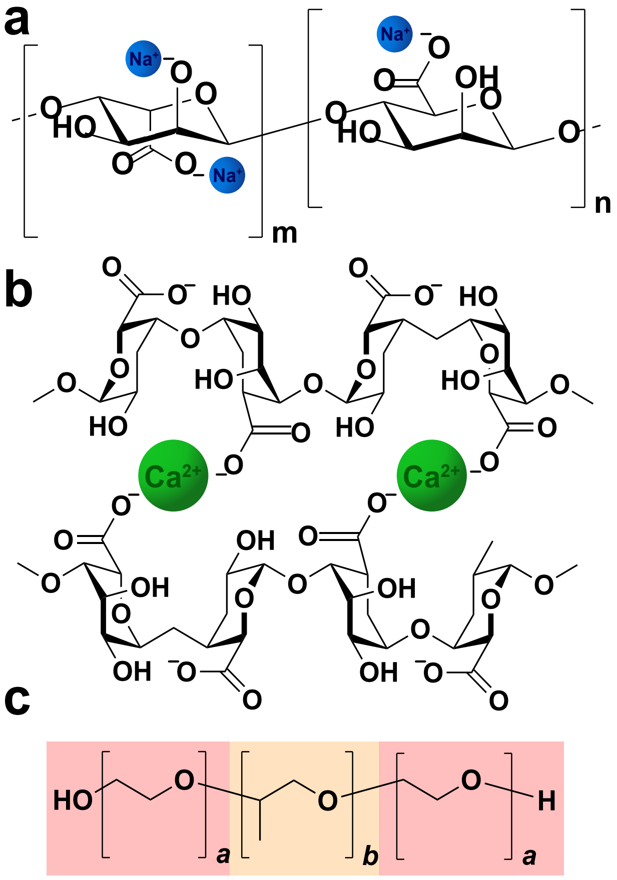

Supplementary Figure 1. *Chemical structures of the polymers used to make porous hydrogels*. **a** Linear alginate polymer comprised of (1→4)-linked β-D-mannuronate (m) and α-L-guluronate (n), bound to monovalent sodium ions (Na^+; blue spheres). **b** Guluronate chains of alginate cross-linked by divalent calcium ions (Ca^{2+}; green spheres). **c** Structure of the triblock copolymer poloxamer 407, comprised of hydrophilic polyethylene glycol (red boxes) and hydrophobic propylene glycol (orange box). *a* = 101 and *b* = 56.

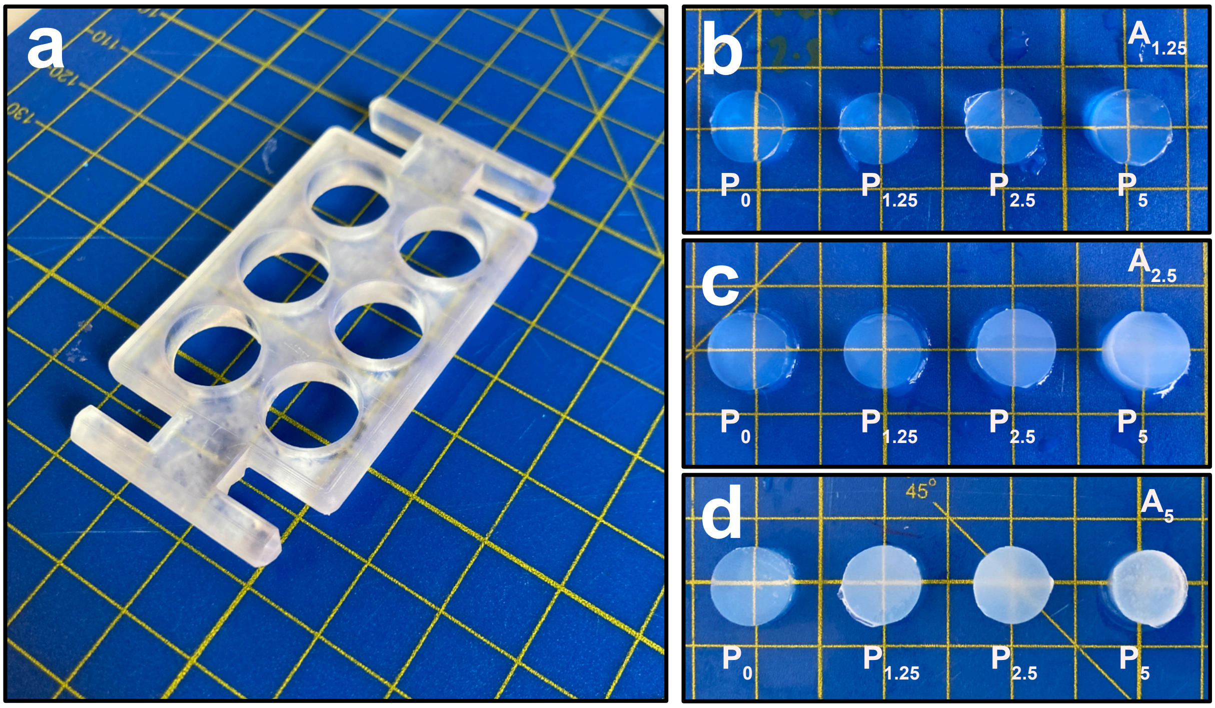

Supplementary Figure 2. *Forming hydrogels for compression testing*. **a** The mold used in dialysis to yield hydrogel discs 12 mm in diameter and 5 mm in height. **b** Optical images of the $A_{1.25}$ hydrogel discs with increasing Poloxamer concentration. **c–d** Corresponding images of the $A_{2.5}$ and A_5 hydrogels, respectively. Yellow lines demarcate 1 cm.

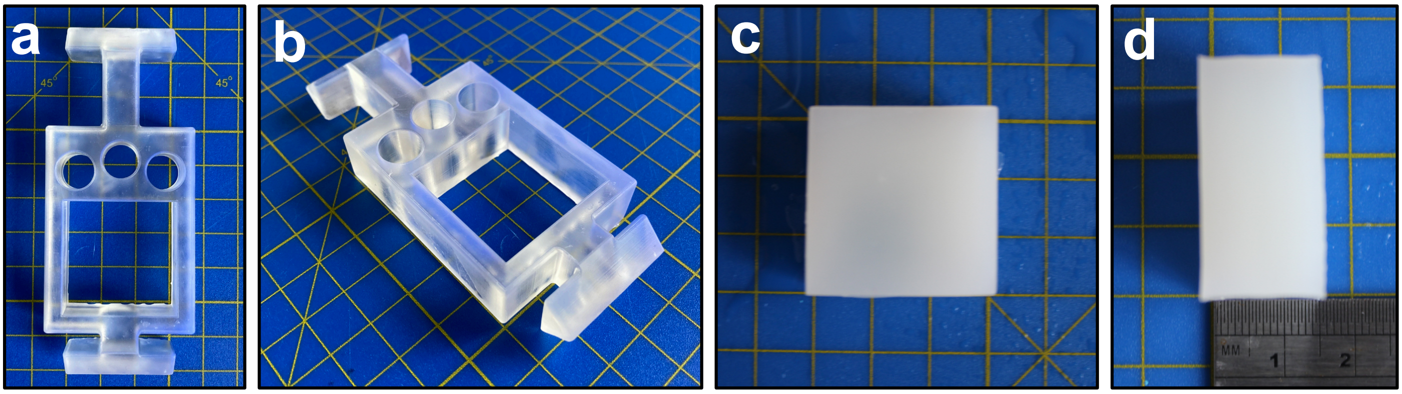

Supplementary Figure 3. *Forming the hydrogels for dynamic mechanical characterisation*. **a–b** Optical images of the mould used in dialysis to form hydrogel blocks 30×30×15 mm. **c–d** Images of the blocks.

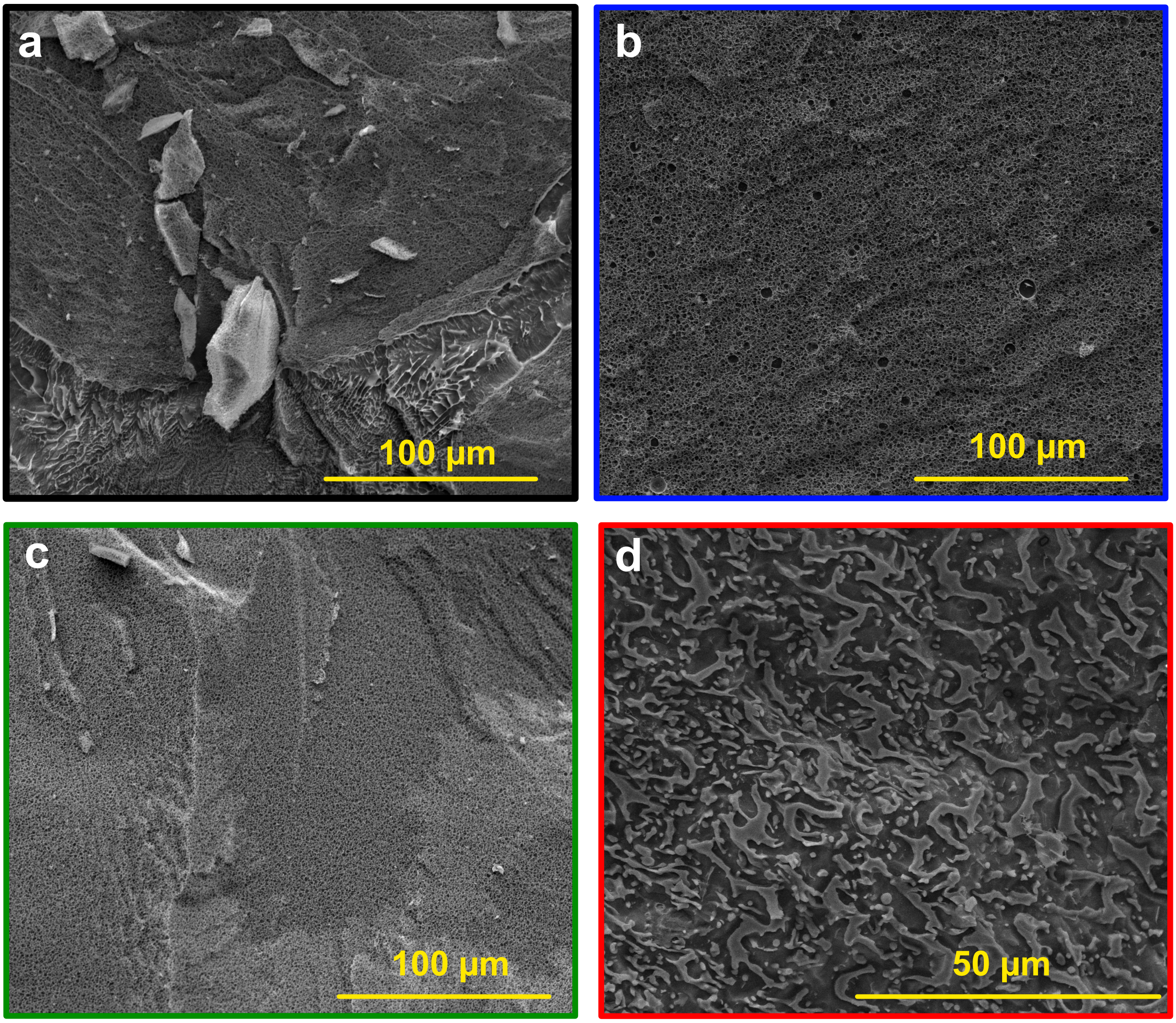

Supplementary Figure 4. *Overviews of cryo-EM fracture surfaces.* **a** A_5–P_0 hydrogel; **b** A_5–$P_{2.5}$ hydrogel; **c** A_5–P_5 hydrogel; and **d** A_5–P_{10} hydrogel. Chunks or debris in the image are a result of the fracture process, not features of the hydrogel.

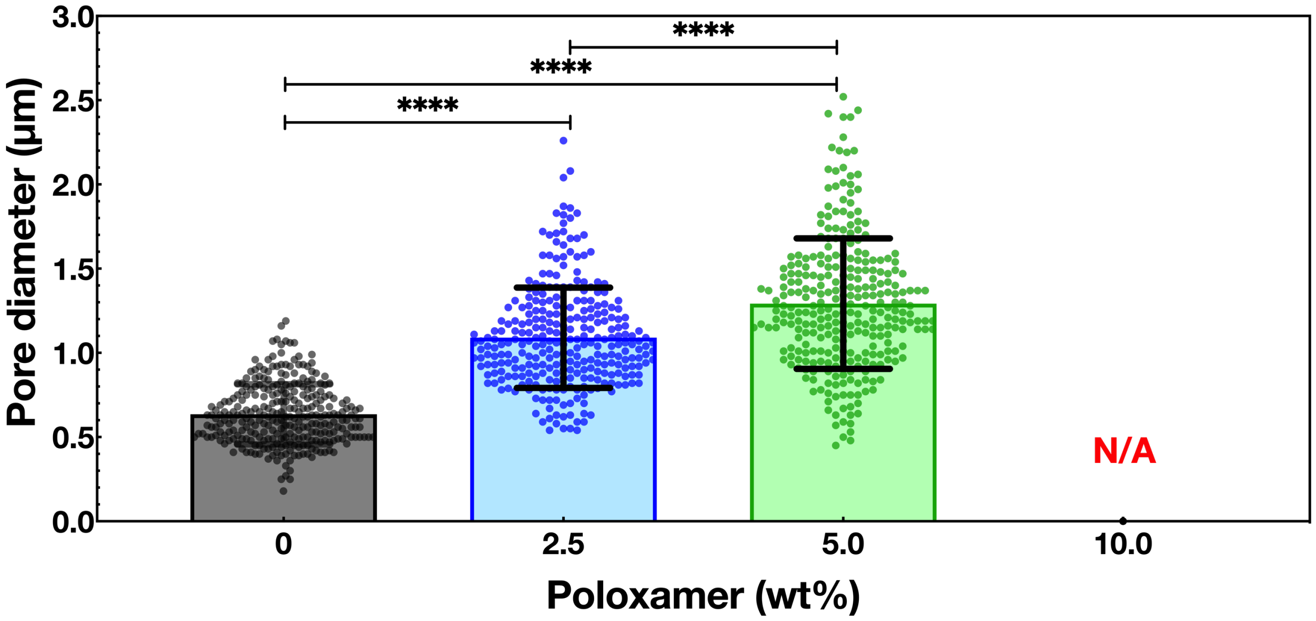

Supplementary Figure 5. *Pore diameters of the A_5 hydrogels.* Average pore diameter and distribution measured from the cryo-EM micrographs. n = 290, error bars = SD. **** = p <0.0001.

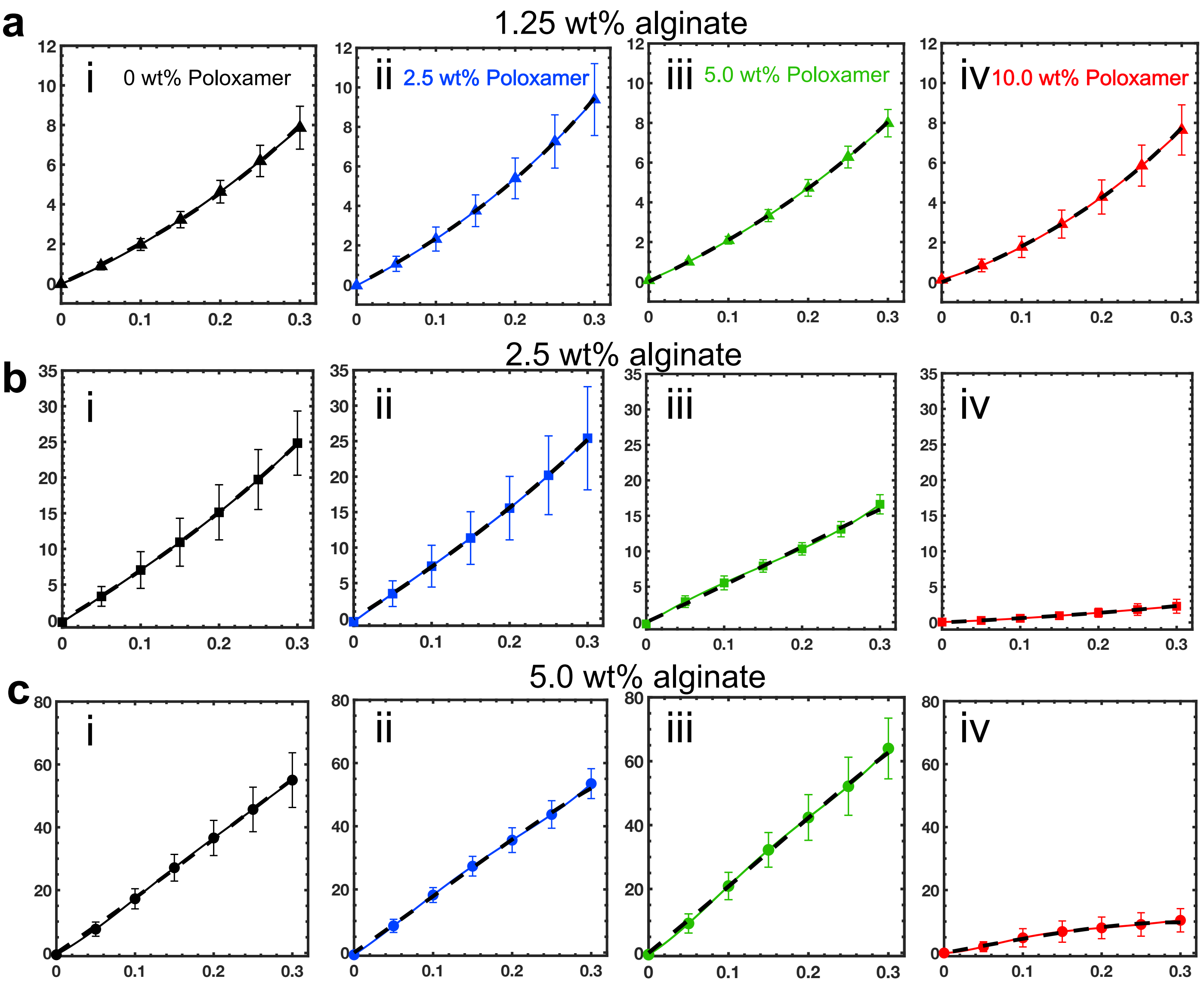

Supplementary Figure 6. *The quasi-static uniaxial compression data was fitted to a Mooney–Rivlin model of hyperelasticity.* **a** $A_{1.25}$ hydrogels; **b** $A_{2.5}$ hydrogels; **c** A_5 hydrogels at (i) 0 wt% black; (ii) 2.5 wt% blue; (iii) 5 wt% green; and (iv) 10 wt% red poloxamer concentrations. Solid lines with symbols indicate the experimental data and the dashed lines indicate the Mooney–Rivlin model. Error bars indicate range.

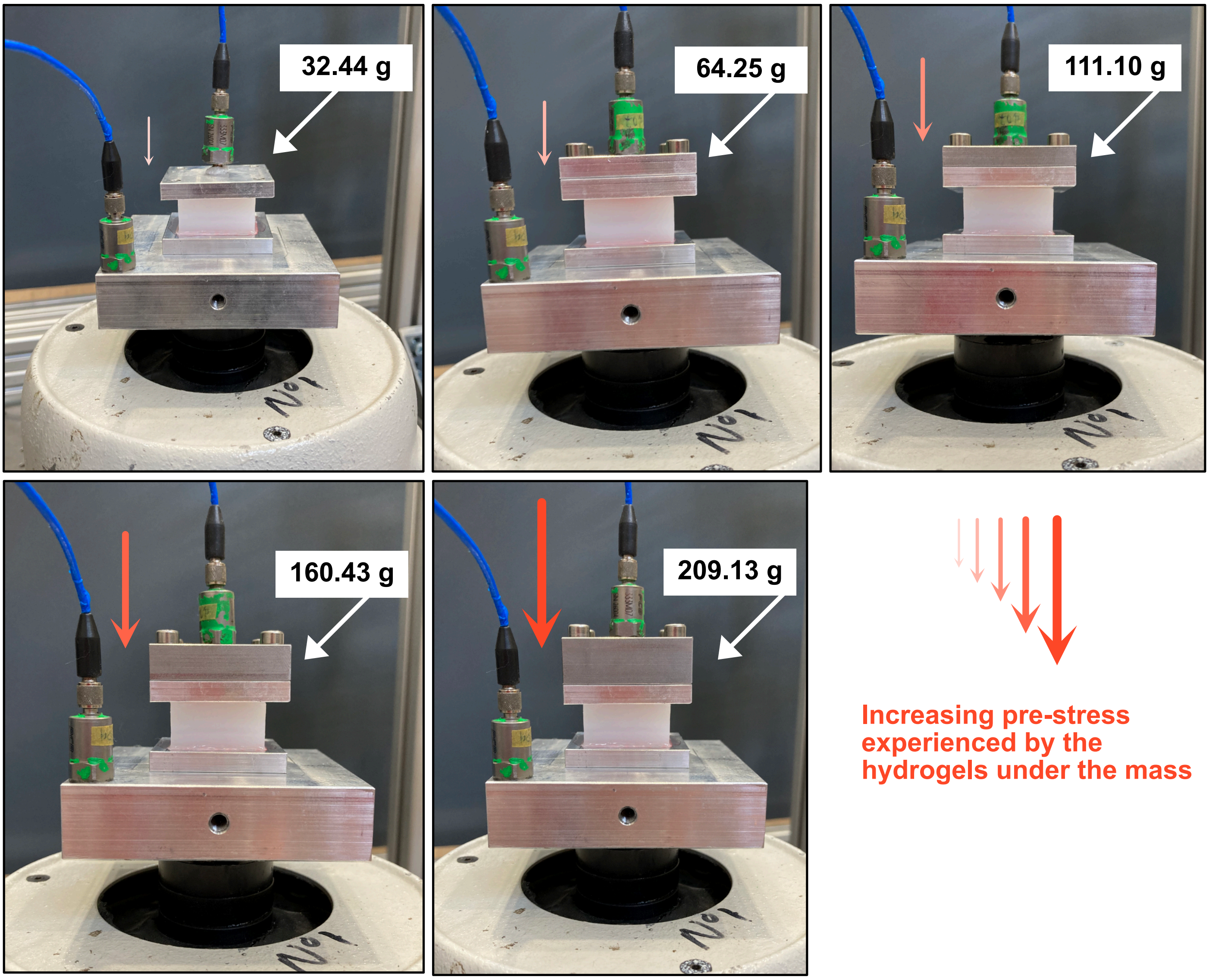

Supplementary Figure 7. *Photos of the vibration transmissibility set-up.* The top mass increases, as indicated, increasing the pre-stress experienced by the hydrogel sample (red arrows).

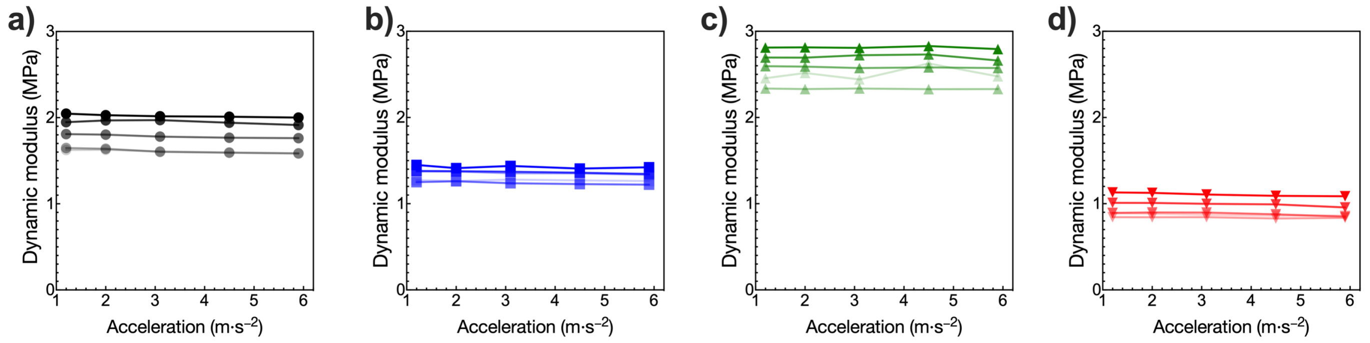

Supplementary Figure 8. *Linearity of the dynamic mechanical analysis set-up.* The dynamic modulus of A_5–P_0 (**a**; black), A_5–$P_{2.5}$ (**b**; blue), A_5–P_5 (**c**; green), and A_5–P_{10} (**d**; red) at increasing acceleration rates under increasing degrees of pre-stress (increasing opacity).

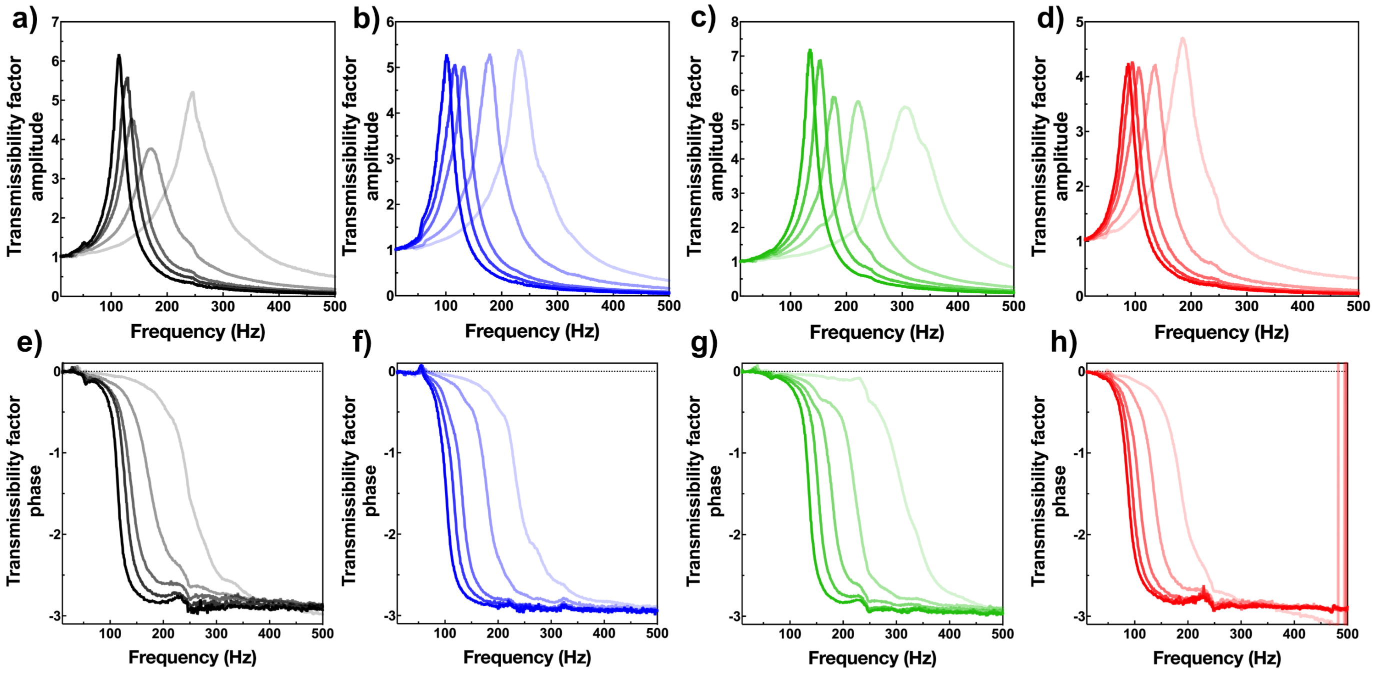

Supplementary Figure 9. Transmissibility factor amplitudes (**a–d**) and corresponding phase curves (**e–h**) of A_5–P_0 (black), A_5–$P_{2.5}$ (blue), A_5–P_5 (green), and A_5–P_{10} (red). Increasing opacity corresponds to increasing pre-stress. This data is also displayed in Figure 5b, but re-plotted here per individual hydrogel composition.

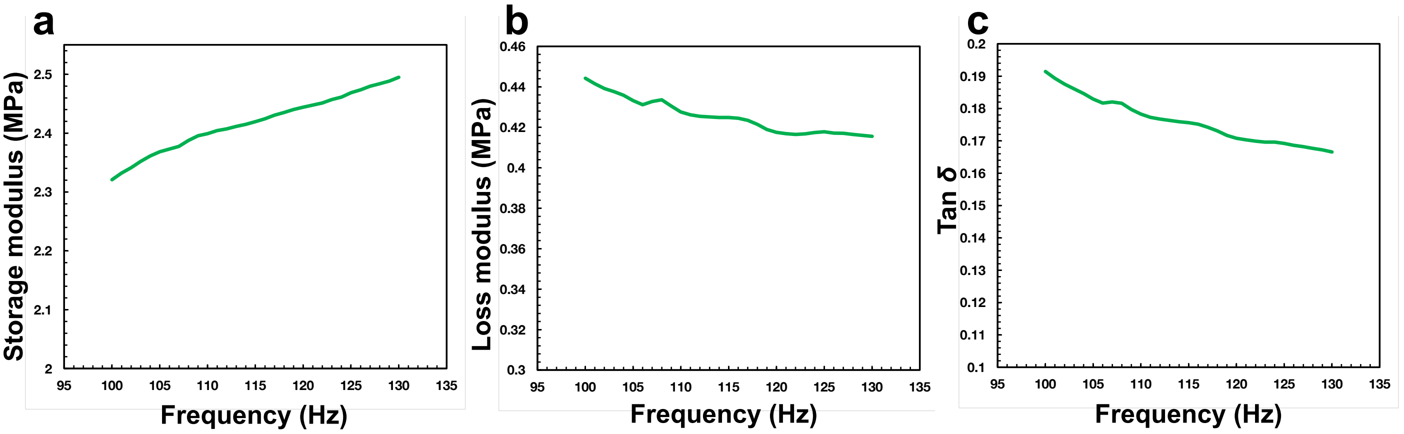

Supplementary Figure 10. *Characterization of A₅–P₅ hydrogel using a dynamic mechanical analyzer (DMA) between 100–130 Hz*. **a** Storage modulus. **b** Loss modulus. **c** Tan δ. Tests were done at 25 °C, with a pre-loading force of 0.001 N and oscillation amplitude of 10 μm.

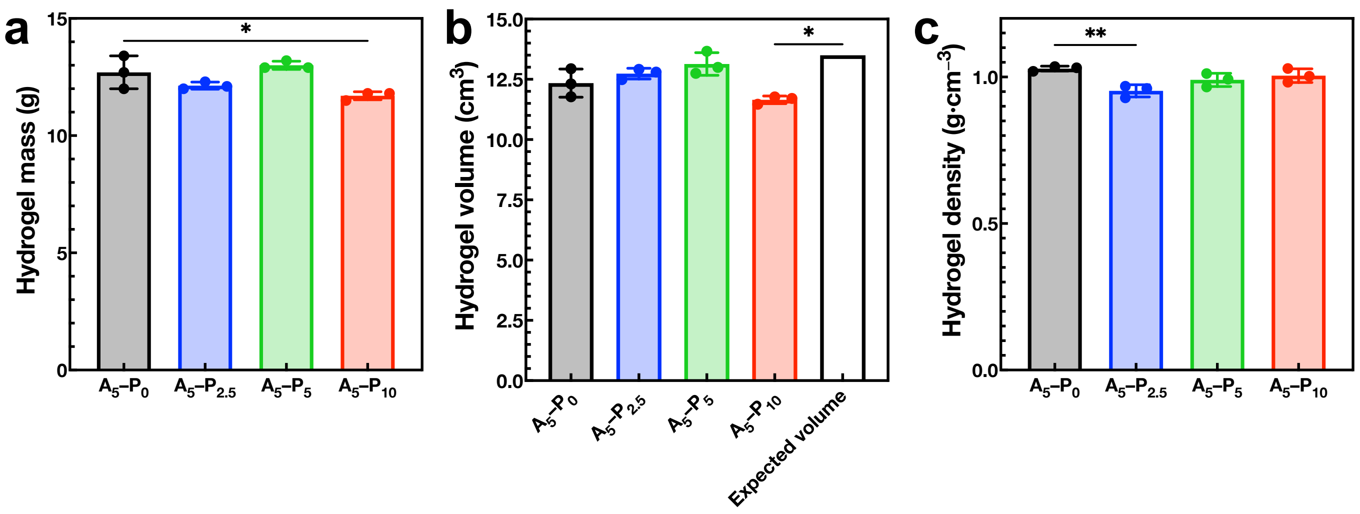

Supplementary Figure 11. *Form information of the hydrogel blocks subject to vibration transmissibility experiments.* **a** Mass of the hydrogel blocks; **b** volume of the hydrogel blocks, the expected volume was derived from the volume in the mold used to produce the blocks; and **c** the density of the hydrogel blocks.

Supplementary tables

Supplementary Table 1. The mass of alginate, Poloxamer 407, and water required to make 50 g gels for dialysis casting.

Hydrogel	Alginate mass (g)	Poloxamer 407 mass (g)	Water mass (g)
$A_{1.25}$–P_0	0.625	0.0	49.375
$A_{1.25}$–$P_{2.5}$		1.25	48.125
$A_{1.25}$–P_5		2.5	46.875
$A_{1.25}$–P_{10}		5.0	44.375
$A_{2.5}$–P_0	1.25	0.0	48.75
$A_{2.5}$–$P_{2.5}$		1.25	47.5
$A_{2.5}$–P_5		2.5	46.25
$A_{2.5}$–P_{10}		5.0	43.75
A_5–P_0	2.5	0.0	47.5
A_5–$P_{2.5}$		1.25	46.25
A_5–P_5		2.5	45.0
A_5–P_{10}		5.0	42.5

Supplementary Table 2. Derived coefficients from the Mooney–Rivlin model of hyperelasticity.

Alginate (wt%)	Poloxamer (wt%)	C_{01} (kPa)	C_{10} (kPa)
1.25	0.0	3.17	-0.13
	2.5	3.41	0.08
	5.0	3.67	-0.47
	10.0	2.03	0.60
2.5	0.0	14.81	-3.90
	2.5	16.15	-4.72
	5.0	14.31	-5.87
	10.0	1.02	-0.11
5.0	0.0	45.75	-17.61
	2.5	52.06	-22.87
	5.0	58.18	-24.36
	10.0	17.72	-9.87
