# Supplementary material for: Tunable network architecture in a hydrogel with extreme vibration damping properties
Source: Commun Mater. 2025 Jul 11;6(1):148. doi: 10.1038/s43246-025-00857-5 (PMC12254035; doi:10.1038/s43246-025-00857-5)
Supplement: Supplementary file 2 — Description of Additional Supplementary files [file 43246_2025_857_MOESM2_ESM.pdf]

Description of Additional Supplementary Files

File name: Supplementary Movie 1

Description: Video of an example vibration transmissibility experiment. The increasing pitch in sound corresponds to five base acceleration rates analyzed.
